# Intra-oral myofascial therapy versus education and self-care in the treatment of chronic, myogenous temporomandibular disorder: a randomised, clinical trial

**DOI:** 10.1186/2045-709X-21-17

**Published:** 2013-06-05

**Authors:** Allan Kalamir, Petra L Graham, Andrew L Vitiello, Rodney Bonello, Henry Pollard

**Affiliations:** 1 Department of Chiropractic, Faculty of Science, Macquarie University, North Ryde, NSW, Australia; 2 Department of Statistics, Faculty of Science, Macquarie University, North Ryde, NSW, Australia; 3 Department of Academic Affairs, Anglo-European College of Chiropractic, Bournemouth, Dorset, UK; 4 School of Exercise Science, Faculty of Sports Science, Australian Catholic University, Sydney, NSW, Australia

**Keywords:** Myofascial pain syndrome, Trigger points, Craniomandibular disorders, Temporomandibular joint dysfunction syndrome, Musculoskeletal manipulations, Exercise, Education, Self-care, Clinical trial

## Abstract

**Background:**

Myogenous temporomandibular disorders (TMD) are considered to be a common musculoskeletal condition. No studies exist comparing intra-oral myofascial therapies to education, self-care and exercise (ESC) for TMD. This study evaluated short-term differences in pain and mouth opening range between intra-oral myofascial therapy (IMT) and an ESC program.

**Methods:**

Forty-six participants with chronic myogenous TMD (as assessed according to the Research Diagnostic Criteria Axis 1 procedure) were consecutively block randomised into either an IMT group or an ESC group. Each group received two sessions per week (for five weeks) of either IMT or short talks on the anatomy, physiology and biomechanics of the jaw plus instruction and supervision of self-care exercises. The sessions were conducted at the first author’s jaw pain and chiropractic clinic in Sydney, Australia. Primary outcome measures included pain at rest, upon opening and clenching, using an eleven point ordinal self reported pain scale. A secondary outcome measure consisted of maximum voluntary opening range in millimetres. Data were analysed using linear models for means and logistic regression for responder analysis.

**Results:**

After adjusting for baseline, the IMT group had significantly lower average pain for all primary outcomes at 6 weeks compared to the ESC group (p < 0.001). These differences were not clinically significant but the IMT group had significantly higher odds of a clinically significant change (p < 0.045). There was no significant difference in opening range between the IMT and ESC groups. Both groups achieved statistically significant decreases in all three pain measures at six weeks (p ≤ 0.05), but only the IMT group achieved clinically significant changes of 2 or more points.

**Conclusion:**

This study showed evidence of superiority of IMT compared to ESC over the short-term but not at clinically significant levels. Positive changes over time for both IMT and ESC protocols were noted. A longer term, multi-centre study is warranted.

**Trial registration:**

Australian and New Zealand Clinical Trials Registry ACTRN12610000508077.

## Background

Temporomandibular disorder (TMD) treatment trends in recent decades have leaned toward multi-modal as well as multi-disciplinary management, in line with that of other chronic musculoskeletal conditions [1]. Such strategies often suggest the use of less invasive and reversible interventions, and has been mainly represented by the involvement of psychotherapy (utilising technology such as biofeedback [2], cognitive and behavioural therapies [3,4]); physiotherapy [5-9] (utilising exercises, mobilisation and various electro-medical therapies); and complementary and alternative medicine therapies (chiropractic [10,11], osteopathy [12,13], massage [14-17], relaxation therapy [18], acupuncture [19,20] and others [21-25]). This trend away from more invasive and irreversible treatment is also represented by an increase in the literature pertaining to the use of patient education as well as self-care (relaxation, implementation of cognitive and behavioural therapeutic strategies and exercises) [26].

In the particular case of the various physical therapies, references are broadly made to manual therapy (mobilisation, manipulation, massage) [14,27,28] and also increasingly to self-care activities (less well defined in the literature, but has been reported to include heat packs, self-massage, active range of motion exercise, isometric exercise, and passive self-mobilisation) [29-31]. The more integrated model of treatment in these therapies [6,28] has resulted in a lack of data on the comparative benefits of the various component interventions [29].

The use of intra-oral myofascial therapies (IMT), such as “trigger point releases” has been well entrenched in a wide variety of physical therapy professions, particularly manual medicine, chiropractic, physiotherapy, osteopathy and massage. The authors have previously published the results of a clinical trial comparing IMT to a combined IMT, education and self-care protocol [32,33]. That trial’s results suggested that combining education and self-care (ESC) with IMT did not appear to afford any significant superiority in pain outcomes in the short term when compared to IMT alone. The authors therefore decided to directly explore the clinical effectiveness of ESC as a stand-alone therapy as compared to IMT over the short term (6 weeks) utilising similar primary outcome measures of pain at rest, upon opening and clenching (i.e. an 11 point ordinal self reported pain scale). A secondary outcome measure of inter-incisal opening range measured in millimetres was also adopted for the study.

In this paper, we present the results of a short-term randomised clinical trial, conducted within a suburban Sydney chiropractic and TMD clinic, comparing outcomes in pain and opening range in dentist-referred participants suffering from chronic myogenous TMD.

## Methods

### Design

The study was part of a PhD program undertaken by the first author in the Faculty of Science at Macquarie University, N.S.W., Australia. The design was that of a randomised trial comparing two different conservative care modalities- IMT and ESC. The trial was conducted in accordance with the CONSORT statement, and was registered with the Australian and New Zealand Clinical Trials Registry on the 21st of June 2010, registration number ACTRN12610000508077. The trial was approved by the Macquarie University Human Ethics Committee on the 10th of August 2010, Reference number 5201000771.

### Study setting

The trial was conducted at the first author’s private TMD and chiropractic clinic in Edensor Park, NSW, Australia. Participants were recruited by referral from several co-operative local dental clinics that already had a well established history of inter-referral and co-management of TMD patients.

### Study team

The trial team consisted of a receptionist, an assistant, one practitioner and an assessor. The receptionist answered telephone queries, verbally discussed basic inclusion and exclusion criteria with enquirers, made appointments and prepared files. The assistant was tasked with generating the randomisation schedule using a web-based random number generator and allocate each numbered participant file to one of the two groups until the schedule was exhausted. This schedule was kept off premises by the assistant, who was blinded to the assessments. Group allocation was concealed from all personnel except for the assistant before randomisation. The practitioner role was undertaken by the first author, who performed the interventions. The assessor was previously trained in the administration of the Research Diagnostic Criteria (RDC) for TMD assessment, using video footage as well as practice drills, in order to calibrate for variables such as pressure, location and participant instruction. All baseline and outcome data were collected on-premises by the assessor, who was blinded to the group allocation of participants. The first author was also blinded to the assessment outcomes until the end of the entire data collection.

### Subjects

Recruitment occurred between August 2010 and February 2011. Interested parties were invited to phone the clinic for further information and to establish basic inclusion and exclusion criteria. Inclusion criteria consisted of an age restriction between 18 and 50 years old, a daily history of peri-auricular pain (with or without joint sounds) for at least the last three months, and voluntary participation. Participants were not remunerated for their participation. Exclusion criteria screened by the receptionist included the use of dentures; a history of malignancy in the last five years; other physical contra-indications such as active inflammatory arthritides, fractures, dislocations, known instability of the jaw or neck; metabolic , connective tissue, haematologic and rheumatologic diseases.

Enquirers who met these requirements then attended the clinic in person to read and sign their consent forms and to have their baseline assessment. The RDC has been reported to be a valid and reliable bi-axial diagnostic tool for the assessment of myogenous, arthrogenous and mixed trait TMD and is widely used in TMD research [34-39]. The RDC contains both a physical axis of assessment as well as a psychosocial one that was applied to establish specific inclusion criteria which included: a myogenous TMD diagnosis (mixed trait and arthrogenous TMD diagnoses were excluded) and a minimum ordinal pain scale score of 3/10 on each of the three symptom outcome measures included in the study. A further exclusion criterion based on the assessment was a finding of severe depression or somatisation on the psychosocial assessment axis.

### Outcome measures

The primary outcome measures used in this trial consisted of the difference between the IMT and ESC groups for each of the three pain measures: jaw pain at rest; jaw pain upon maximal active opening and jaw pain upon clenching. It was hypothesised that these three positional pain measures would give a reasonable interpretation of myofascial pain when the jaw elevator muscles are at a resting physiological tone, undergoing maximal active stretch and maximum isometric contracture. The use of a self reported eleven point numerical rating scale (where zero means “no pain” and 10 represents “pain as bad as could be”) provided ease of use for participants, they having familiarised themselves with ordinal pain scales during the administration of the RDC. A difference of 2 or more points between the groups was considered clinically significant, based on previously published studies [32,33].

A secondary outcome measure was that of the difference between groups for maximal voluntary inter-incisal opening range in millimetres, with an increase in opening distance being considered positive. The use of opening range as an outcome measure has been widely reported in the literature, with good support for both its validity and reliability [40] compared to other movements such as lateral deviation, protrusion, retrusion and end-feel stretch pain in these ranges. A 5 mm or more difference between groups was deemed to be clinically significant for measured inter-incisal opening range [41,42].

Interest was also in determining whether each treatment group had declined by a clinically significant about over time for each outcome measure. Reductions in pain of two or more points or increases in opening range of at least 5 mm were deemed clinically significant.

Outcomes were measured during attendance at the clinic at baseline and at six weeks post treatment.

### Group allocation

Each consecutively numbered participant file was allocated to a treatment group according to a blocked design randomisation schedule, which was web-generated (http://www.randomizer.org) and kept off-premises by the assistant.

### Interventions

Participants were randomised into one of two treatment groups, IMT or ESC. Each treatment group received two sessions per week for five weeks. The treatments are described as follows:

1. IMT group, whose treatment consisted of several myofascial techniques previously reported in the literature [32,33], and administered by the first author. They were comprised of the following three interventions:

**Figure 1:**
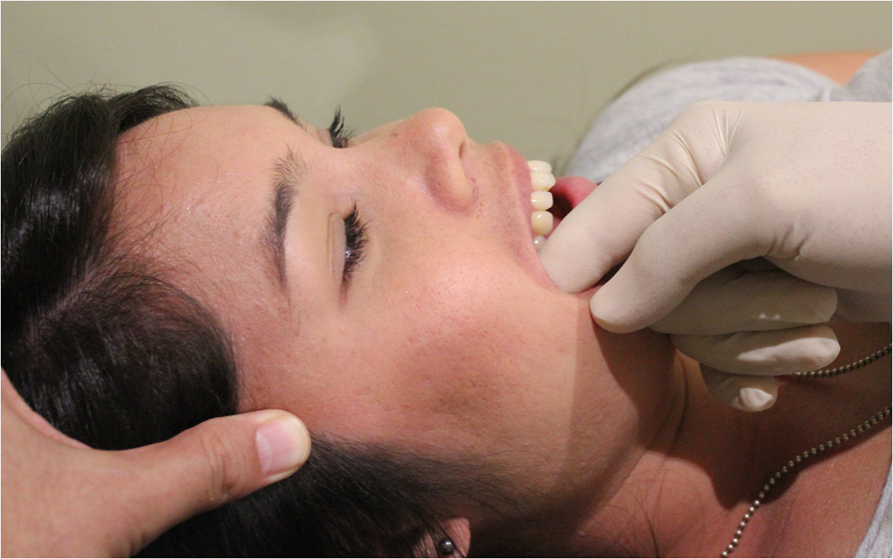
Intra-oral temporalis release.

**Figure 2:**
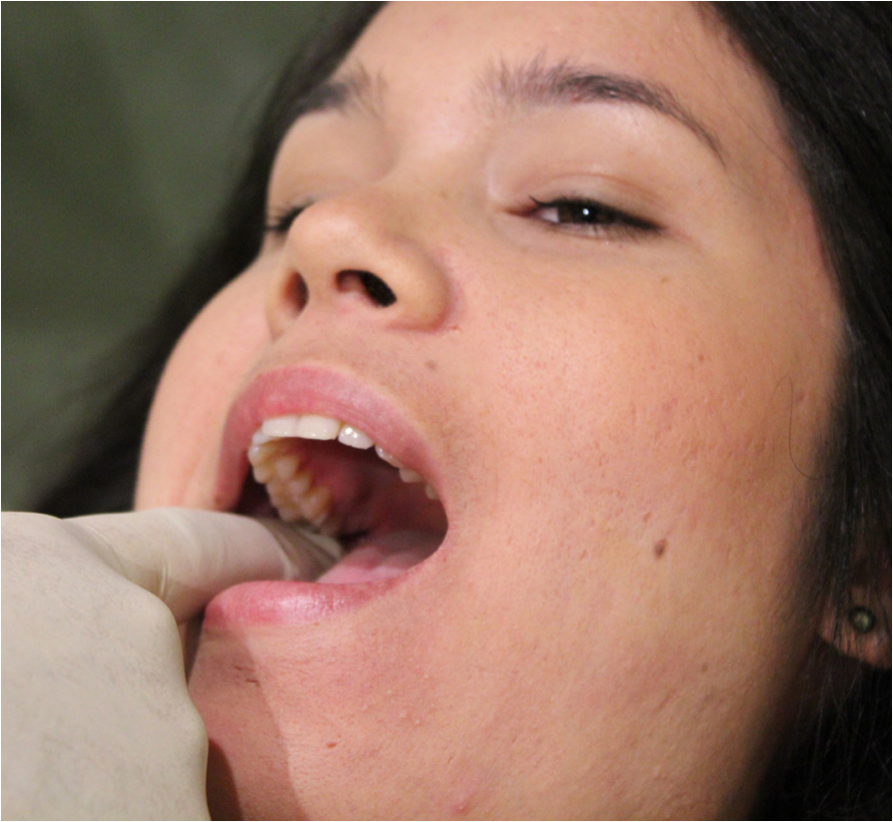
Intra-oral medial and lateral (origin) technique.

**Figure 3:**
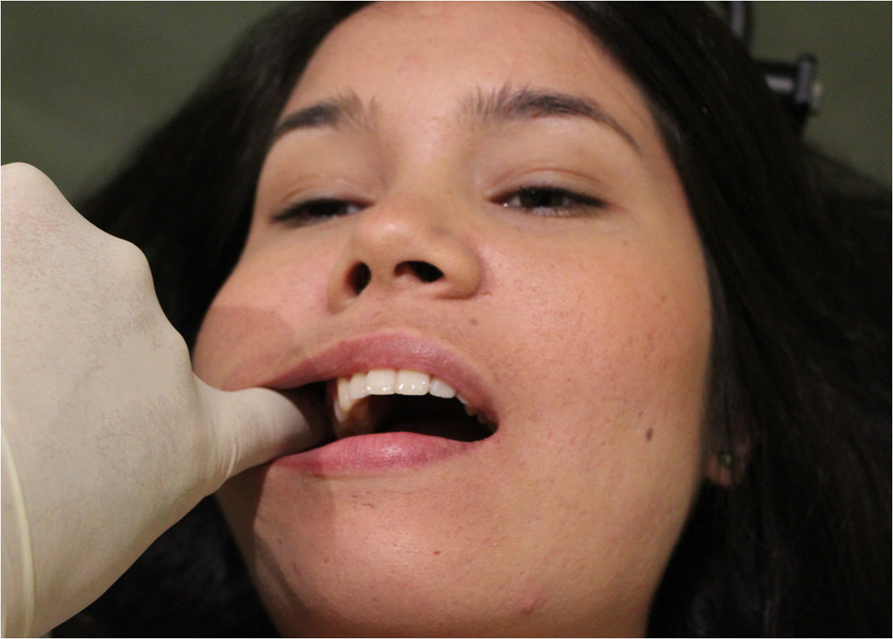
Intra-oral sphenopalatine ganglion technique.

a) “Intra-oral temporalis release” (Figure 1). This consisted of a gloved index finger intra-oral contact onto the tendonous insertions of the temporalis muscle at the superior aspect of the coronoid process. Light posterior and caudad pressure is applied by the finger within pain tolerance of the patient. Simultaneously, the index and middle fingers of the other hand apply superior pressure longitudinally along the anterior fibres of the temporalis muscle moving gradually anterior to posterior. The patient is asked to incrementally open their mouth to its maximum range.

a) “Intra-oral medial and lateral pterygoid (origin) technique” (Figure 2). The practitioner is seated either homolateral or contralateral to the side being treated. A gloved index finger is inserted along the lateral wall of the pharynx, posterior to the last molar. Posterior and cephalad pressure is applied into the pharyngeal mucosal tissues overlying the pterygoid origins arising from the lateral pterygoid plate of the sphenoid. Care is taken to avoid direct contact of the hamulus. The contact is maintained for 5 seconds.

a) “Intra-oral sphenopalatine ganglion technique” (Figure 3). The gloved 5th finger of the caudad hand is slowly inserted along the buccal surface of the lightly occluded teeth. The patient is asked to briefly clench their teeth, and upon relaxing, the practitioner presses their finger deeper posteriorward. This process is repeated until the tip of the finger reaches as close to the anterior aspect of the infratemporal fossa / sphenopalatine fossa as is comfortable to the patient. The patient is then asked to lift their head off the table, pushing into the contact. In this way excessive force by the practitioner is checked by an apprehension response of the patient. After three repetitions, the patient relaxes; resting their head back onto the headrest, and gentle buccal pressure is now applied into medial pterygoid muscle by the practitioner’s finger tip before gently being removed from the mouth.

2. ESC group, whose treatment was based on the protocol also previously described by the authors in the literature [32,33], consisted of short scripted lectures on the basic anatomy, biomechanics and pathophysiology of the TMJ, the role of stress; slow, diaphragmatic breathing exercises and general advice on relaxation awareness and avoidance of potentially problematic foods (nuts, chewing gum etc.). This component was partially based on the prior published work by Michelotti et al. [43]; Nicolakis et al. [44] and Dworkin [45]; with further recent work by Jerjes et al. [46]. These ESC sessions also involved the teaching and supervision self-care exercises that were performed both during the session, to ensure proper form, as well as at home by the participant twice a day. The same number of attendances at the clinic and duration of sessions were given to this group. The exercises, which are designed to stimulate and stretch the joint capsule and relax the masticatory muscles are summarised below:

**Figure 4:**
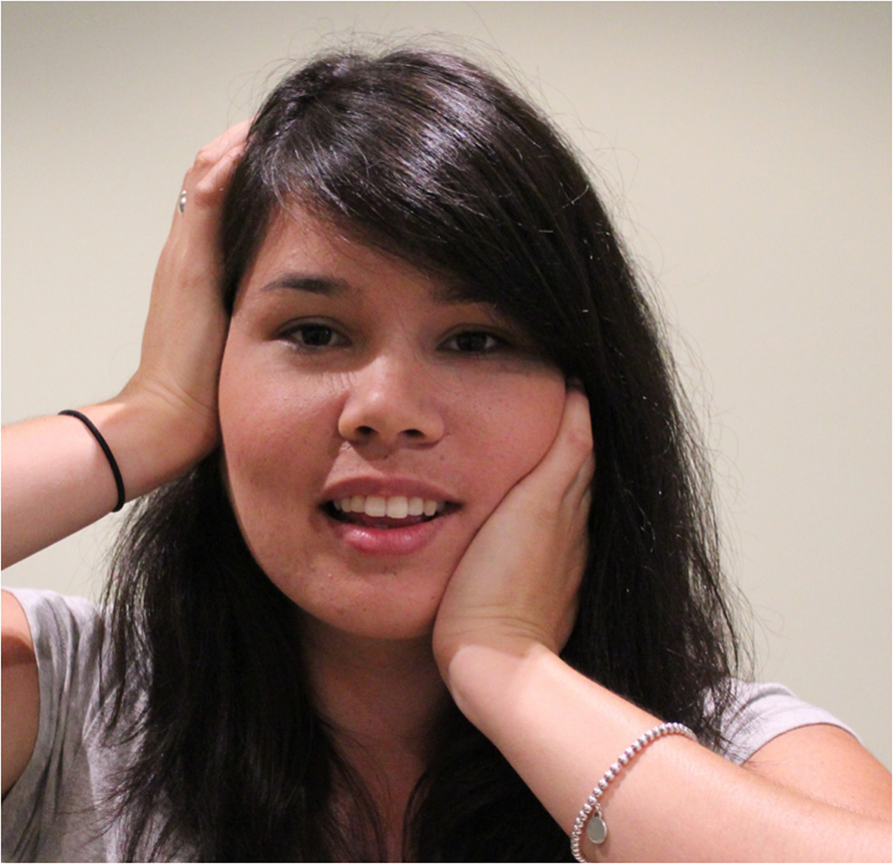
Guided and controlled jaw excursions.

**Figure 5:**
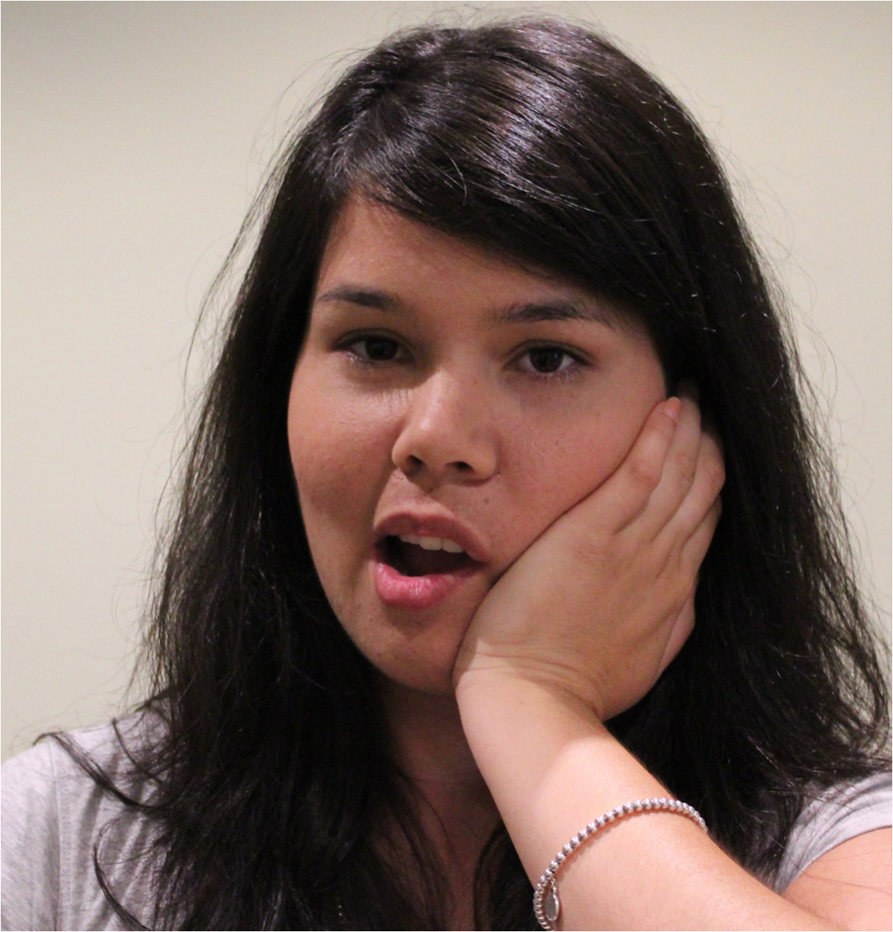
Post-isometric stretches (lateral deviation).

**Figure 6:**
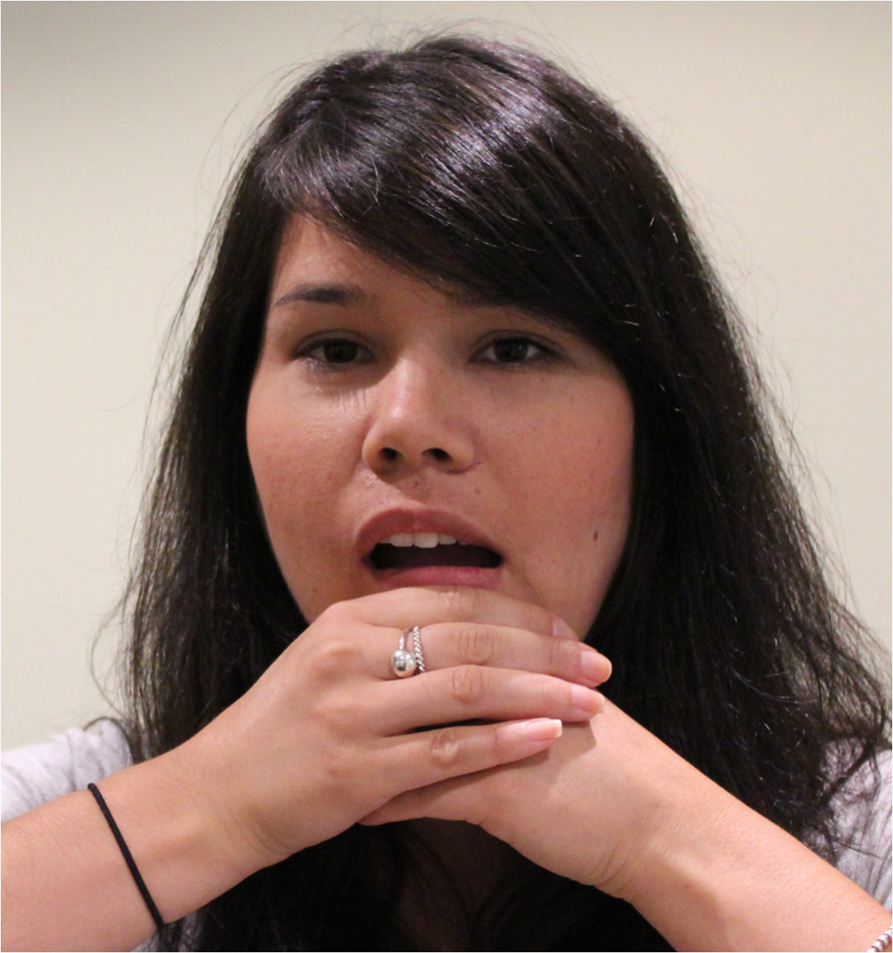
Post-isometric stretches (opening).

a) Guided and controlled jaw excursions (Figure 4). The patient applies a contact to the TMJ joint of one side with the thenar or pisiform of the ipsilateral hand, while the heel of the other hand is placed on the side of the chin. Both sides exert even pressure upon their contacts, while the patient actively opens and closes their mouth five times. Where tolerable, the patient may increase the pressure exerted by the hand contacts with each successive opening. The contacts are then reversed and repeated on the other side.

a) Post –isometric stretches (lateral deviation and opening, Figures 5 and 6). Placing the heel of one hand on the same side of the chin, the patient exerts and active force of the chin into the hand, which opposes any movement. The contraction is held for up to 10 seconds, depending on the tolerance of the patient. The contraction is then relaxed, while the hand continues to exert some pressure into the chin, deviating it slightly towards the other side. The cycle of isometric contraction is continued at this new point, and repeated in increments until the jaw has deviated to its tolerable limit. The contacts are then reversed and the procedure repeated on the opposite side. A similar process is then applied to incremental opening of the jaw, which is achieved by cupping the chin with both hands and resisting an isometric jaw close contraction, then drawing the jaw into an incrementally greater opening distance.

### Statistical analysis

The data were analysed using linear regression models with pain outcome score at the end of the study as the dependent variable and baseline score as a covariate together with treatment group. For the primary outcomes results are presented as adjusted mean follow-up score with standard deviation and a p-value for the between groups contrast from the associated model. A Bonferroni correction was used whereby a significance level of 0.017 was used to reflect the three comparisons made in the primary outcome (i.e. 0.05 divided by 3).

The secondary outcome measure, opening range in mm, was also analysed using a linear regression model as described above. Results are presented as average adjusted difference in opening range (mm) between groups together with 95% confidence interval (CI).

Remaining within group differences for the pain measures and opening range measurements were presented as the mean change over time with 95% CIs as estimated from a linear model of change over time against treatment group.

Based on the a priori determination of clinical significance, an additional analysis was undertaken whereby for each outcome, change over time was coded as a 1 (success) if it had reached a clinically significant change or better and otherwise it was coded as 0 (failure). Logistic regression was then used with the new binary variable as the outcome and treatment group and baseline score as covariates. Estimated odds of success for IMT versus ESC are presented for each outcome with 95% CI.

Sample size was estimated according to data published by Dao et al. [47]. Setting the significance level to be 0.05 and the power at 80%, Dao estimated that a 60% difference in pain intensity between groups would require a sample of approximately 42 participants (i.e. a group size of 21 participants per group, depending on the number of groups). The enrolment goal was set to 46 participants, in order to account for a possible ten percent drop-out rate.

For an average two point difference in pain measures (noted earlier as a clinically relevant difference) between the treatment groups using a 0.017% level of significance, power of 80% and estimated standard deviation between groups taken from the main trial [32], only 9 participants would be needed for each group. The larger number initially estimated was retained to increase the power to detect differences between the groups.

The models were fitted using R version 2.15.0. [48].

## Results

**Figure 7:**
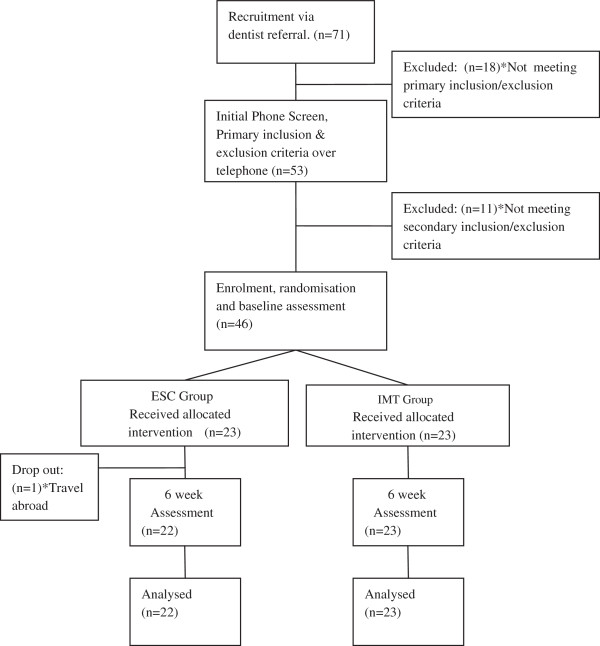
Study flow-chart.

The study flow chart is presented in Figure 7. Recruitment of participants commenced in August 2010 and concluded in February 2011. There were 71 enquiries, based on local dentist referrals of which 53 met the basic requirements and qualified for an assessment. Of those, 46 met their specific inclusion and exclusion criteria and were consecutively enrolled into the study as participants according to the randomisation schedule, having signed their consent forms. All of the participants accepted their group allocation. Treatments commenced subsequent to baseline assessment and were completed through February 2011. The last of the post-treatment assessments were completed by the end of April 2011. The interventions were successfully administered and the trial concluded without any reports of adverse reactions in any participants. One participant dropped out of the ESC group before the second assessment citing work-related travel prohibiting their continued treatment.

Baseline data, presented in Table 1, showed some differences in baseline scores with the IMT group having higher average pain scores and greater opening range.

**Table 1:** Background demographics and baseline characteristics of the participants

**Detail**	**ESC group**	**IMT group**
	**(n = 23)**	**(n = 23)**
	**Mean (SD)**	**Mean (SD)**
Age in years	26.8 (6.81)	28.2 (9.43)
Gender (m:f)	8:15	9:14
Pain^§^ at rest	4.09 (0.90)	4.74 (1.36)
Pain^§^ on opening	4.57 (1.24)	5.17 (1.47)
Pain^§^ on clenching	5.00 (1.28)	5.87 (1.58)
Opening range mm	37.43 (4.14)	38.83 (4.98)

*ESC* Education and self-care, *IMT* Intra-oral myofascial therapy, *SD* Standard deviation, *§* Self reported 11 point ordinal pain scale.

### Primary outcomes

Adjusted post treatment results for the pain scores are shown in the final two columns of Table 2. Results indicate strong evidence of a statistically significant difference between groups however this difference was not clinically significant.

**Table 2:** Average adjusted pain scores at 6 weeks and difference between groups

**Variable**	**ESC: Mean**	**IMT: Mean**	**IMT v ESC**	**P-value**
	**(SD)**	**(SD)**	**(98.3% CI)**	
RP	2.87 (0.37)	2.26 (0.55)	−0.88 (−1.44, -0.31)	<0.001
OP	3.22 (0.59)	2.35 (0.70)	−1.16 (−1.80, -0.53)	<0.001
CP	3.39 (0.46)	2.61 (0.57)	−1.10 (−1.81, -0.38)	<0.001

*RP* Pain at rest, *OP* opening pain, *CP* Clenching pain, *ESC* education and self-care group, *IMT* intra-oral myofascial therapy group, *SD* standard deviation, *P-value* and 98.3% *CI* (confidence interval) is for the between groups difference at six weeks adjusted for baseline score.

### Secondary outcome

Results for opening range showed that, at six weeks, the average adjusted difference between groups in opening ranges was not significant (0.66, 95% CI:-0.96, 2.29; p = 0.416). The mean opening range for both the IMT and ESC groups had increased from baseline (p = 0.032 and 0.025, respectively, Table 3). However, from the point of view of clinical significance, neither the between groups nor within groups changes achieved a minimum range change of at least 5 mm post treatment.

**Table 3:** Average change in pain and opening range over time with 95% confidence interval

**Variable**	**ESC (6 weeks v BL)**	**IMT (6 weeks v BL)**
RP	−1.22 (−1.64, -0.80)	−2.48 (−2.90, -2.06)
OP	−1.35 (−1.81, -0.89)	−2.83 (−3.29, -2.37)
CP	−1.61 (−2.15, -1.06)	−3.26 (−3.81, -2.72)
OR	2.52 (1.37, 3.67)	3.00 (1.85, 4.15)

*RP* Pain at rest, *OP* opening pain, *CP* Clenching pain, *OR* Opening range (mm), *ESC* education and self-care group, *IMT* intra-oral myofascial therapy group, *BL* baseline.

For the pain outcomes, Table 3 demonstrates that both groups achieved statistically significant reductions in pain for all three outcome measures at six weeks compared to baseline. The IMT group achieved a clinically significant reduction of at least 2 points for each of the three pain outcomes whereas the ESC group did not.

Table 4 shows that the odds of achieving a two-point decrease was significantly higher for IMT versus ESC for resting pain and similarly for opening pain and clenching pain, respectively although the intervals were very wide. Odds of a achieving a 5 mm increase in opening range was not significantly different for IMT versus ESC.

**Table 4:** Proportion of successes and estimated adjusted odds of success IMT versus ESC

**Variable**	**ESC: Proportion of successes**	**IMT: Proportion of successes**	**IMT v ESC**	**P-value**
			**OR (95% CI)**	
RP	6/23	19/23	27.65 (4.21, 417.52)	<0.001
OP	9/23	19/23	9.90 (1.92, 78.37)	0.012
CP	10/23	19/23	5.20 (1.12, 30.27)	0.045
OR	6/23	5/23	0.84 (0.20, 3.37)	0.805

*RP* Pain at rest, *OP* opening pain, *CP* Clenching pain, *OR* Opening range, *ESC* education and self-care group, *IMT* intra-oral myofascial therapy group, *SD* standard deviation, *CI* confidence interval.

## Discussion

The results showed that a TMD trial of this nature can be successfully conducted within a private suburban physical therapy clinic, where there is good co-operation with, and referral from local dentists. There was only one drop-out from this short study, which was not typical of other trials that have variably reported rates of up to 30% [43,49]. Studies of drop-out causes have suggested reasons such as environmental obstacles, perceived improvement or dissatisfaction with treatment [43,50] for that figure. The local and de-institutionalised nature of the treatment and assessment facilities and trust in their dentist’s referral by patients may account for the good retention rate in this study. In addition, the chronicity; rigorous minimum pain scale and inclusion criteria [51]; benign nature of the interventions, and short duration of the trial may also have contributed.

The main outcome analysis showed that baseline adjusted pain scores were significantly lower for IMT compared to ESC however the difference was not clinically significant over this short term trial. Despite this failure to achieve clinical significance, a secondary responder analysis suggested significantly higher odds of the IMT group achieving a clinically important two-point decrease over the ESC group.

Clinical significance has been described in various ways in the literature. The breadth of change is often considered to be an important indicator, with simplified interpretations of self reported 11 point ordinal measures for pain intensity scales suggesting a 2 point shift as being clinically significant [52-54]; and at least a 5 mm shift in opening range scale measures being considered clinically significant [41,42]. A limitation of this study is that it did not employ a comprehensive evaluation of post-treatment disability and patient satisfaction as advocated in TMD guidelines [55].

In light of this, fully evaluating clinical significance means that other factors also need to be taken into account, such as the importance of the change to patients as well as the efficiency of treatment and cost to consumers [56]. A study encompassing broader and more comprehensive outcome measures may be useful and should be conducted over a longer time frame.

The changes observed in pain scores over the course of the trial suggested improvement in pain for both IMT and ESC over the short term though only IMT reached clinically significant improvements. The IMT techniques employed in this study have a long history of being associated to craniomandibular myofascial trigger points- particularly within the chiropractic, osteopathic and physiotherapy / physical medicine professions although the added improvement in this group may be attributable to environmental or other factors not investigated in this study.

Active myofascial trigger points cause clinically perceivable pain complaints, and are tender to palpation. They refer recognizable pain upon contraction, and when compressed, produce referred motor and/or autonomic phenomena. They are also thought to contribute to muscle tension and decreased range of motion [57].

In this study, in spite of the positive trend, there was neither a significant difference between groups nor a clinically significant change in maximum active opening range in either group. It has been suggested that maximal pain free opening range benefits more from a combined treatment approach (such as combining IMT and ESC) than through individual treatment modalities alone [43]. The results of a previous trial by the authors to that effect concur with this idea [32]. However, the modest range of opening findings in this trial may just reflect its short time scale, or just the nature of the participants sampled in this study.

The improvement observed in the ESC group may be explained by several factors. It is thought that the effects of explaining the benign nature of the condition in detail, as well as providing reassurance, are powerful tools for the remission of TMD symptoms [43]. Carefully structured, simple interventions that emphasise self-care are also thought to be of significant benefit to TMD sufferers [58], as are enforcing patient responsibility and simultaneously addressing control factors [59]. Of course, the improvement in this group may also be attributable to factors beyond the ESC therapy that were not considered in this study.

This study was primarily hampered by the limitation that for a chronic condition, it was run over a short time frame (six weeks). It was also run from a single centre, using a single practitioner. This makes generalisation difficult. However, the positive short term results should encourage further, more comprehensive research into patient education and self-care for TMD. They also provide some additional support for the use of IMT protocols already published by the authors.

## Conclusion

This study demonstrated significantly lower mean pain scores for IMT versus ESC treatment approaches in myogenous TMD sufferers and significantly higher odds of IMT achieving a two or more point decrease in pain scores over ESC therapy. Both treatments indicated positive effects over time however the short duration of the trial suggests that the results should be interpreted with caution. In light of these findings, we suggest that any further research into myofascial and self-care strategies for TMDs (of any type) use trials of at least one year duration to assess potential benefit.

## Competing interests

The authors declare that they have no competing interests.

## Authors’ contributions

AK being the PhD candidate was involved in the conception, design, ethics and registration, interventions, tabulation, statistical analysis, manuscript write-up. PG was involved in supervising the statistical analysis and manuscript review. AV was involved in the conception and manuscript review. RB was involved in the ethics and manuscript review. HP was involved in the conception of the study and its design. All authors read and approved the final manuscript.
